# Identification of responsive genes to multiple abiotic stresses in rice (*Oryza sativa*): a meta-analysis of transcriptomics data

**DOI:** 10.1038/s41598-024-54623-7

**Published:** 2024-04-01

**Authors:** Mahnaz Azad, Masoud Tohidfar, Rahele Ghanbari Moheb Seraj, Mohammad Mehralian, Keyvan Esmaeilzadeh-Salestani

**Affiliations:** 1 Department of Cell & Molecular Biology, Faculty of Life Sciences & Biotechnology, Shahid Beheshti University, Tehran, 19839-69411 Iran; 2 Department of Horticultural Sciences, Faculty of Agriculture and Natural Resources, University of Mohaghegh Ardabili, Ardabil, Iran; 3 Department of Agriculture, Medicinal Plants and Drugs Research Institute, Shahid Beheshti University, Tehran, 19839-69411 Iran; 4 Chair of Crop Science and Plant Biology, Institute of Agricultural and Environmental Sciences, Estonian University of Life Sciences, Kreutzwaldi 1, 51006 Tartu, Estonia

**Keywords:** Meta-analysis, Microarray, Rice, Environmental stresses, qRT-PCR, Biotechnology, Plant sciences

## Abstract

Abiotic stresses limit the quantity and quality of rice grain production, which is considered a strategic crop in many countries. In this study, a meta-analysis of different microarray data at seedling stage was performed to investigate the effects of multiple abiotic stresses (drought, salinity, cold situation, high temperature, alkali condition, iron, aluminum, and heavy metal toxicity, nitrogen, phosphorus, and potassium deficiency) on rice. Comparative analysis between multiple abiotic stress groups and their control groups indicated 561 differentially expressed genes (DEGs), among which 422 and 139 genes were up-regulated and down-regulated, respectively. Gene Ontology analysis showed that the process of responding to stresses and stimuli was significantly enriched. In addition, pathways such as metabolic process and biosynthesis of secondary metabolites were identified by KEGG pathway analysis. Weighted correlation network analysis (WGCNA) uncovered 17 distinct co-expression modules. Six modules were significantly associated with genes involved in response to abiotic stresses. Finally, to validate the results of the meta-analysis, five genes, including *TIFY9 (JAZ5), RAB16B, ADF3, Os01g0124650,* and *Os05g0142900* selected for qRT-PCR analysis. Expression patterns of selected genes confirmed the results of the meta-analysis. The outcome of this study could help introduce candidate genes that may be beneficial for use in genetic engineering programs to produce more tolerant crops or as markers for selection.

## Introduction

Rice (*Oryza sativa*) is one of the world's most important cereals and a staple food for half of the world's population^1^. Since the global population is rapidly growing and is predicted to reach 9.9 billion by 2050^2^, the production of rice must increase at least 1% annually to meet its demand^3^. On the other hand, environmental stresses have been primarily considered as the factors that reduce the quantity and quality of agricultural products. Abiotic stresses including drought, salinity, low and high temperatures, deficiency of essential nutrients, and accumulation of heavy metals can negatively affect the growth, development, and yield of the crops. Abiotic stresses annually reduce rice production by 32% (about three million tons) in the world^4^. Therefore, to improve sustainability, there is a need to increase the yield in breeding programs by introducing stress-tolerant varieties.

During the evolution of plants, different mechanisms including various physiological, cellular, and molecular modifications have been developed to cope with different stresses and to survive in adverse conditions^5^. Abiotic stresses significantly affect physiological processes such as flowering, grain filling, and maturation. It has been reported that abiotic stresses affect plant metabolisms including photosynthesis, enzyme activity, mineral nutrition intake, and respiration^6^. Plants respond to abiotic stresses by inducing a complex network of genes. They activate stress-related genes to adapt to new environmental conditions through the perception and transduction of stress signals^7^. Sensing, signaling, transcription, transcript processing, translation, and post-translational protein modifications are plant molecular mechanisms to respond to abiotic stresses^8^. The thickness of the cell wall, production of reactive oxygen species (ROS), and secretion of phytohormones including abscisic acid (ABA), jasmonic acid (JA), salicylic acid (SA), and ethylene (ET) are other defense responses of plant to abiotic stresses^9^.

To better understand the complex system of molecular processes and identify pathways and mechanisms involved in cell response to abiotic stresses, it is inevitable to use statistical and computational approaches. High-throughput technologies such as microarray and RNAseq, which are being used for gene expression analyses, have made it feasible to study a large number of genes simultaneously in different conditions. With the development of new technologies, a big step has been taken to decipher the gene regulatory networks in plants' stress-tolerance mechanisms.

Meta-analysis is a standard statistical procedure for combining datasets from multiple studies to systematically assess previously published data to derive more comprehensive conclusions about that research field^10^. This technique provides a broad perspective on specific biological questions and more reliable results than individual studies^11,12^.

Different studies have identified several responsive genes under abiotic stresses in rice through omics data analysis such as genomics and transcriptomics^13–17^. In a meta-analysis, de Abreu Neto et al. investigated the genes involved in redox homeostasis in rice under abiotic stresses and showed that only 4% of differentially expressed genes (DEGs) were in the ROS mechanism pathway directly^13^. ROS plays an important signaling role in plants, especially in response to biotic and abiotic stimuli^14^. Another meta-analysis reported by Cohen et al. investigated different abiotic stresses including drought, salinity, high and low temperature as well as biotic stresses such as Dwarf, Stripe, ZB13, and Guy11 viruses, and Xoc and Xoo bacteria in rice^15^. They showed that the number of DEGs varied from 1220 to 11,644 in different experiments, in which 5863 and 2154 genes were common in all abiotic and biotic stresses, respectively. Buti et al. investigated different responses of susceptible and tolerant genotypes of rice under osmotic, chilling, and salt stresses. They found 35 hub genes through gene network analysis, which 24 of them were located in at least one known QTL of rice such as qLRC-1, qGY-2b, qTGW-2a, rfw1b, rfw4a, qtl3.1, gpl11.1, gw11.1, yld11.1, rn3 and qSDW2 which are related to cold, drought, and salinity^16^. Recently, Ramkumar et al. identified 6657 multiple abiotic stress-responsive genes (salinity, drought, and heat stresses) in rice at the seedling stage. They found 10 modules containing 10 genes through gene network analysis that were common to all three studied stresses^17^.

In the present study, a large-scale meta-analysis was performed to integrate different microarray studies focused on abiotic stresses including drought, salinity, cold and high temperatures, alkali conditions, nutrients (nitrogen, phosphorus, and potassium) deficiency, toxicity of heavy metal, iron, and aluminum to find genes involved in different stresses. We hypothesized that there may be some common genes and pathways that are activated and expressed to alleviate stress and regulate plant metabolisms under different stress conditions. Therefore, it may lead to the introduction of genes and pathways in breeding programs to have resistant cultivars.

## Materials and methods

### Data collection and meta-analysis

Microarray data sets of studied abiotic stresses including drought, salinity, cold and high temperatures, alkalinity, deficiency of essential nutrients such as nitrogen, phosphorus and potassium as well as the toxicity of heavy metals, aluminum, and iron were extracted from Gene Expression Omnibus (http://www.ncbi.nlm.nih.gov/geo) (Table 1). Two technical considerations were applied to select microarray datasets: (1) The selected dataset must be in one of the two subspecies *O. sativa* japonica or *O. sativa* indica; (2) The RNA must have been extracted from the vegetative parts of plants including shoots, roots, and or whole seedlings (reproductive tissues such as seeds, panicles or flowers were excluded from the study).

**Table 1:** Transcriptomics raw data related to different abiotic stress studies of *Oryza sativa* used for the current meta-analysis.

Stress	GEO ID	No. sample	Sub-family	Genotype name	Sample tissue	Time after stress	Replicates per sample
High temperature	GSE14275	6	Japonica	Zhonghua 11 (ZH11)	Seedling	3 h	3
Drought	GSE93917	12	Japonica	Dongjin	Leaf	15 d	3
Salinity	GSE3053	11	Indica	IR29 and FL478	Shoot	7 d	2 or 3
Al stress	GSE107531	6	Japonica	Zhonghua11(ZH11)	Root	6 h	3
Alkali condition	GSE45724	12	Japonica	Jijing88	Shoot	1 d	3
Cold stress	GSE37940	36	Japonica	C418 and CT IL K354	Shoot	2h, 6h, 12h, 24h, and 48h	3
N starvation	GSE109649	6	Japonica	TNG67	Root	1 h	3
K+ deficiency	GSE37161	18	Japonica	Nipponbare	Root	6 h, 3 d, and 5 d	9
P deficiency	GSE60823	12	Japonica	spx1	Leaf	7 d	3
Fe toxicity	GSE131287	12	Indica	EPAGRI 108 and BR-IRGA 409	Root	3 d	3
Heavy metals	GSE25206	15	Indica	IR-64	Root	1 d	3

The method used for meta-analysis of microarray data is based on Raw Data Integration. It integrates raw microarray data from multiple studies through the following steps:

1. Data preprocessing: Extract raw microarray data (e.g., CEL files) from individual studies (raw microarray data from individual studies retrieved from NCBI).
2. Quality control: Perform quality control checks for each dataset to identify and remove low-quality samples or datasets. The quality of each dataset was controlled by checking the boxplot of datasets.
3. Normalization: The normality of each dataset was checked.
4. Data integration: Merge normalized data into a unified dataset. All data series matrices are merged into one dataset. Treated samples (regardless of the type of stress) are categorized into the "stress" group, and all untreated samples are grouped into the "control" group.
5. Batch effect correction: Address batch effects using the ComBat technique to ensure comparability between stress and control samples. The batch effect, as one of the major technical variations that make differences between different datasets, was removed by the SVA R package (v 3.38.0)^18^ according to the COMBAT method^19^.
6. Statistical analysis: Conduct statistical analyses (e.g., Differential expression analysis) on the integrated dataset to identify genes that are differentially expressed across conditions or groups (stress samples vs control samples)^20^.

Meta-analysis of transcriptome data sets was carried out by merging expression data matrix in R software. The DEGs between stress and control samples were identified using the *Limma* R package (v 3.48.1)^21^. Genes with an adjusted P value (p-adj) < 0.05 and |log2 fold change| (|log2FC|) ≥ 0.5 were considered as DEGs. Probe IDs of DEGs were used as queries in the DAVID web-based tool (http://david.abcc.ncifcrf.gov/) to annotate them. A schematic workflow summarizing the main stages of the current study is presented in Fig. S1.

### Enrichment analysis

The DEGs were subjected to singular enrichment analysis (SEA) in agriGO (http://bioinfo.cau.edu.cn/agriGO/) to identify enriched gene ontologies (GO). DEGs were classified into three biological process (BP), molecular function (MF), and cellular component (CC) with a significant threshold of p-value < 0.05. The Kyoto Encyclopedia of Genes and Genomes (KEGG) (https://www.genome.jp/kegg/) analysis was performed to identify enriched pathways, in which DEGs are significantly involved.

### Protein–protein interaction network

To identify key genes responsible for abiotic stresses, hub gene determination analysis was performed for all DEGs. Protein–protein interaction (PPI) network was constructed using the STRING database (https://string-db.org/) by submitting DEGs as input. The output file was imported to Cytoscape (version 3.8.2) software for visualization and edition^22^. The CytoHubba plugin and Maximal Clique Centrality (MCC) algorithm were used to identify highly connected genes as hubs^23^.

### Weighted correlation network analysis (WGCNA)

The WGCNA R package was used to identify the group of genes with similar expression patterns under stress situations^24^. The WGCNA is performed as a system biology approach for analyzing the correlation pattern between genes and spreading them into co-expression modules^25^. The co‐expression analysis was performed for paired genes using a Pearson correlation matrix. The weighted adjacency matrix was constructed using the power function (β), and then, transformed into a topological overlap measure (TOM) matrix to assess its connectivity in the network^26^. The clustering dendrogram of the TOM matrix was constructed using the average linkage hierarchical clustering. To obtain the correct module number, a restricted minimum gene number of 30 for each module was set and a threshold of 0.25 to merge similar modules was used. The network was visualized for the two most important modules using the Cytoscape software (version 3.8.2). Each module can lead to a real biological process, so to examine the significance of grouping, gene ontology analysis for each module was performed using the DAVID web-based tool.

### Plant materials and experimental design

The rice seeds (Shiroodi variety; japonica subgroup) were obtained from the Iranian Rice Research Institute (Amol, Mazandaran, Iran). Plant studies comply with relevant institutional, national, and international guidelines and legislation. The surface of the seeds was sterilized using 70% ethanol for 2 min, followed by treatment with 1.5% NaClO_4_ for 1 min. The seeds were washed three times with distilled water to remove the detergents^27^. Seeds were placed on the moistened filter paper in the Petri dishes and incubated for the first 72 h in the dark to germinate. Then, they were transferred to pots filled with pearlite and kept in a 16 h light and 8 h dark photoperiod at 25 ± 2 °C and were irrigated every day. The Yoshida solution was used^28^ after the emergence of seedlings. Samples in the control treatment were kept under mentioned condition (16 h light and 8 h dark photoperiod at 25 ± 2 °C), and the Yoshida solution was renewed every 3 days. Seedlings were exposed to different stress treatments according to Table 2. The root and/or shoot samples of different stress treatments with their respective control treatments were collected, immediately immersed in the liquid nitrogen, and kept at − 80 °C until further analysis.

**Table 2:** Details of different treatments used on rice based on previous studies.

Treatment	Age of plant	Applying treatment	Time of being exposed	Tissue sample	Reference
High temperature	14-day-old	Exposed to 42 °C	3 h	Leaf	^29^
Drought	30-day-old	PEG6000 (20%)	48 h	Leaf	^30^
Salinity	At days 11–13	50 mM NaCl	48 h	Shoot	^31^
	At days 13–15	100 mM NaCl	48 h		
	At days 15–23	140 mM NaCl	48h		
Al stress	12-day old	450 µM AlCl_3_	8 h	Root	^32^
		pH = 4.5			
Alkali condition	7-day-old	50 mM (NaHCO_3_ = 9:Na_2_CO_3_ = 1)	24 h	Shoot	^33^
		pH = 9.25			
Cold stress	3-leaf stage	Exposed to 4 °C	6 h	Shoot	^34^
			48 h		
N starvation	10-day-old	Yoshida without NH_4_NO_3_	1 h	Root	^35^
K+ deficiency	14-day-old	Yoshida without K_2_SO_4_	6 h	Root	^36^
			5 d		
P deficiency	14-day-old	Yoshida without NaH_2_PO_4_	7 d	Root	^37^
Fe toxicity	20-day-old	500 mg/l FeSO_4_	3 d	Root	^38^
Heavy Metals	10-day-old	100 µM K_2_Cr_2_O_7_	24 h	Root	^39^

### RNA extraction and cDNA synthesis

Total RNA was extracted using the DENAzist Column RNA Isolation Kit (DENAzist Inc., Mashhad, Iran). The quantity and quality of RNA samples were evaluated by a NanoDrop spectrophotometer and 1% agarose gel electrophoresis, respectively. The first-strand cDNA was synthesized using the EasyTM cDNA synthesis kit Pars Tous according to the manufacturer's instructions (Pars Tous Inc., Mashhad, Iran).

### Validation of abiotic responses of candidate genes by real-time PCR

To validate the reliability of the meta-analysis approach, five genes were randomly selected for real-time PCR. Specific primers were designed by OLIGO Primer Analysis Software v.7.0 (National Bioscience Inc., Plymouth, USA). The optimize the amplification, 10 μl of Taq DNA Polymerase Master Mix RED (Ampliqon, Denmark), 1 μl of cDNA (~ 20 ng), 0.01 µM of each forward and reverse primer, and sterile distilled water (up to 20 μl) were used and PCR products were evaluated on 1% agarose gel for the presence of the desired band and the absence of non-specific amplicons and primers dimer. The optimized PCR program for each gene and oligonucleotide primers are presented in Table 3. Real-time PCR with three technical and five biological replicates was done by the Rotor-Gene Q (QIAGEN, Germany) and SYBR^®^ Green Fluorescent DNA Stain-low ROX (Jena Bioscience, Germany) according to the optimized program for each candidate gene. The elongation factor 1-alpha (*elF1α*) gene of rice (LOC4331813) was selected as the reference gene. The gene expression was calculated using the Delta-Delta CT method^40^ in the REST2009 software according to the comparative threshold cycle, and the graphs were made using the GraphPad Prism9(GraphPad Software, United States).

**Table 3:** List of primers sequences designed to amplify selected genes and reference gene in rice *(Oryza sativa*).

Gene name	Primer sequence	Primer Tm [°C]	Amplicon length (bp)
	F/R		
*TIFY9 (JAZ5)*	F: GTGTGTGTGGTTGTTGCTGTG	70/8	195
	R: TTTGATCGTGAGGCTGACTGC	70/8	
*Os01g0124650*	F: TCCGTCAATAAAACTCGCCC	68/3	116
	R: TGCAGCAAAACACTCTCAAGC	68/9	
*ADF3*	F: AACGAAGGGTTCAAGAAGGAGC	70/8	200
	R: ATCCAAACACCAAGCAAGCCG	70/3	
*RAB16B*	F: CCGGCGAGAAGAAGGGATTC	72/3	175
	R: TTCGAGGACGCTATACACTGC	70/8	
*Os05g0142900*	F: GACAAGGCGTTAGATCATCAG	68/9	203
	R: TTGACTCGACGTTTAAGGAAC	66/9	
*elF1α*	F: TTTCACTCTTGGTGTGAAGCAGAT	70/5	103
	R: GACTTCCTTCACGATTTCATCGTAA	70/9	

### Statistical analysis

Statistical analysis for all molecular data was performed using R version 3.5.321 and RStudio version 1.1.463. Data were analyzed by ANOVA for a completely randomized design with treatments as fixed effects and replicate as random effects. Mean values were compared using the Duncan test function provided in the agricolae package at 5% significance level of probability.

## Results

### Meta-analysis to identify DEGs of rice in response to multiple abiotic stresses

**Figure 1:**
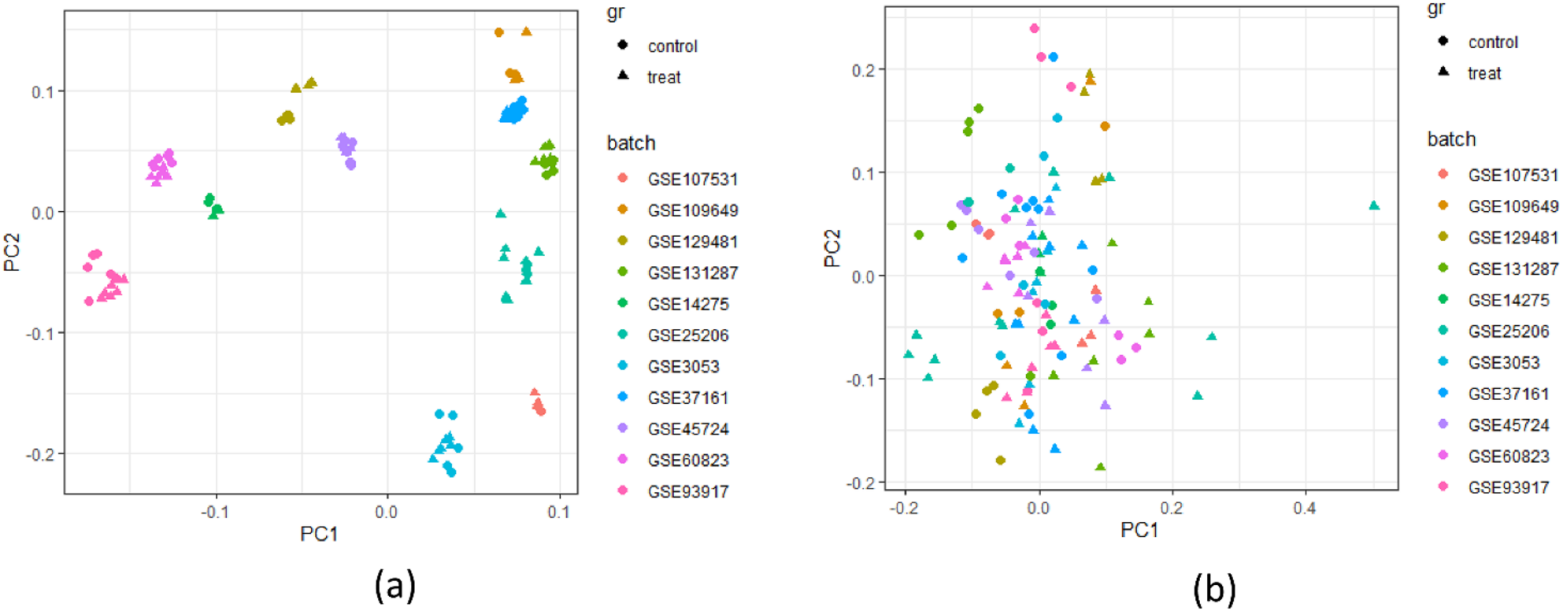
Principle component analysis (PCA) to correct the batch effect. (**a**) PCA before batch effect removal. (**b**) PCA after batch effect removal. Different colors indicated different studies, control and treated samples were indicated by different shapes.

The meta-analysis included 11 studies containing 118 samples. Our result showed 561 DEGs among stressed and control samples, in which 421 and 139 were up- and downregulated, respectively (Table S1). The PCA plot illustrates that the presence of a batch effect leads to the segregation of each dataset based on their respective batches, as indicated by the distinct colors shown in Fig. 1a. However, following the implementation of the ComBat method, the impact of the batch effect is reduced, resulting in the mixing of datasets, regardless of their original batches, as demonstrated in Fig. 1b.

### Gene ontology and KEGG analysis

**Figure 2:**
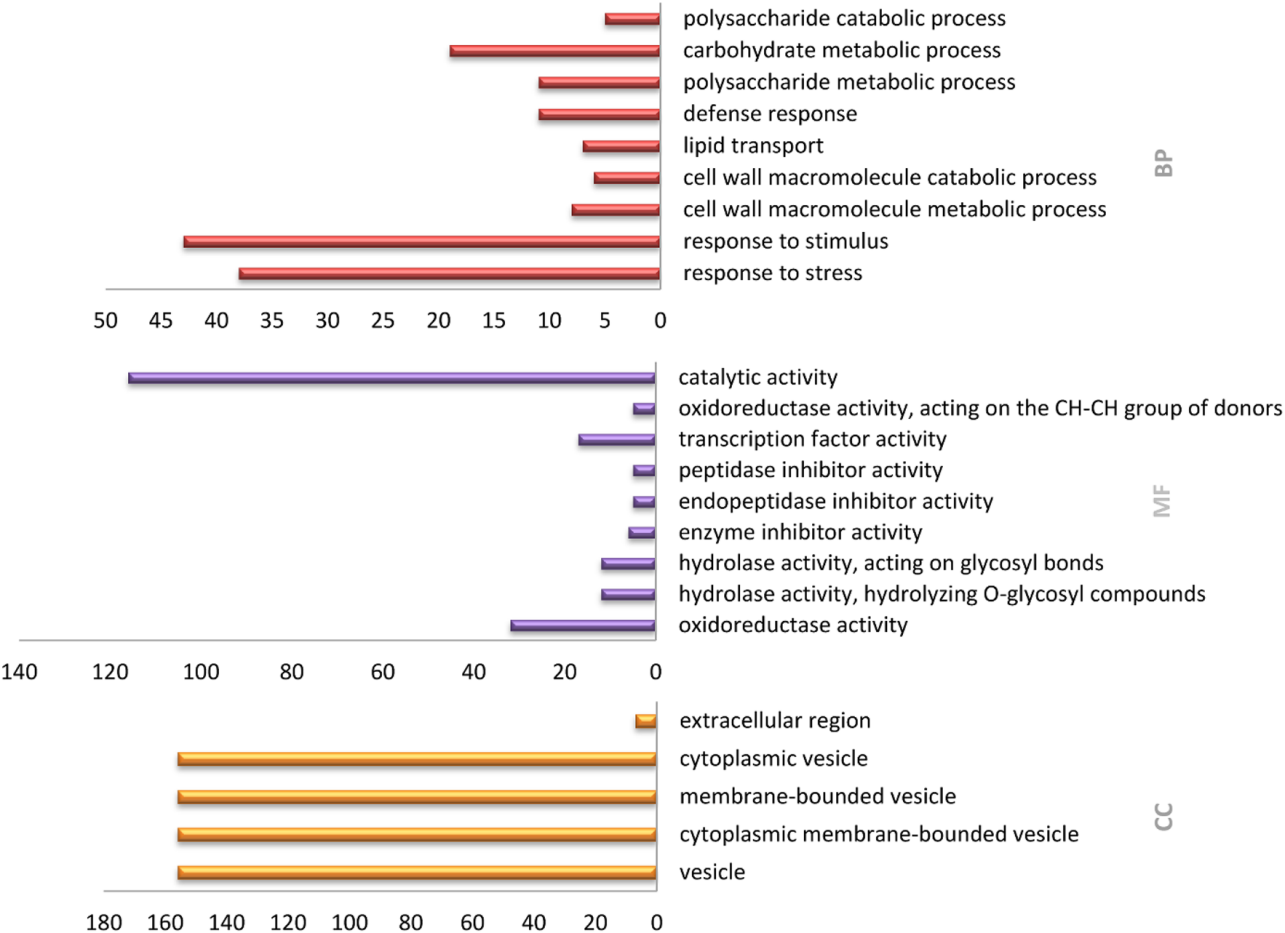
The top enriched GO terms of DEGs (p-value < 0.05) in the categories of BP: Biological Process, MF: Molecular Function, and CC: Cellular Component.

The GO in the biological process associated with DEGs was grouped in 37 terms (p-value < 0.05) (Table S2). The top enriched biological processes were response to stress and stimulus (GO:0006950 GO:0050896), metabolic and catabolic processes of cell wall macromolecules (GO:0044036, GO:0016998), lipid transport (GO:0006869), and defense response (GO:0006952). GO enrichment analyses for functional annotation revealed that oxidoreductase (GO:0016491), hydrolase (GO:0004553, GO:0016798), and enzyme inhibitor (GO:0004857) activities were the top enriched molecular function. In this category, DEGs were grouped in 24 GO (p-value < 0.05) (Table S2) among them there are also regulation activities such as transcription factors (GO:0003700), transcription regulators (GO:0030528), DNA binding (GO:0003677), and enzyme regulator (GO:0030234) activities. Among GO terms in the cellular component, vesicles (GO:0031982), cytoplasmic membrane-bounded vesicles (GO:0016023), membrane-bounded vesicles (GO:0031988), cytoplasmic vesicles (GO:0031410), and extracellular region (GO:0005576) were significantly enriched (Fig. 2).

**Figure 3:**
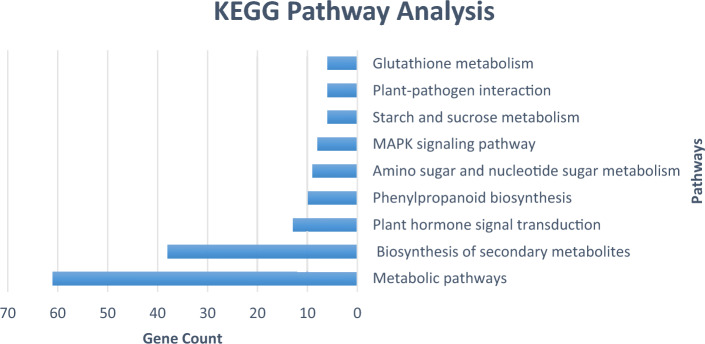
KEGG pathway enrichment analysis. The significant pathway for differentially expressed genes in response to multiple abiotic stresses.

The KEGG analysis showed that DEGs were mostly enriched in the metabolic pathway, biosynthesis of secondary metabolites, plant hormone signal transduction, phenylpropanoid biosynthesis, amino sugar, and nucleotide sugar metabolism, and MAPK signaling pathways (Fig. 3)^41^.

### Identification of DEGs encoding TF and PK in response to abiotic stresses

A total of 25 Transcription factor (TF) genes related to 7 TF families were identified among all DEGs (Table 4). The WRKY family with 8 genes and the Ethylene Response Factor (ERF) family with 6 genes represented the highest number of TFs. Moreover, HSF, MYB, 6HLH and NF-YB factors had 3, 3, 1 and 1 genes, respectively. Protein kinases were encoded by 19 genes that were classified as the receptor-like kinase (RLK) (19 genes) and calcium/calmodulin-dependent protein kinase (CAMK) families (1 gene). As shown in Table 4, the RLK family included 5 subgroups including leucine-rich repeat (8 genes), DLSV (4 genes), and S Domain 2b (4 genes) with the highest number of genes, respectively.

**Table 4:** List of TF families and protein kinase groups identified among DEGs and the number of genes in each family.

TF family	No. of TF	Protein kinase	No. of PK
WRKY	8	RLK (leucine-rich repeat)	8
ERF	6	RLK (DLSV)	4
HSF	3	RLK (S Domain 2b)	4
MYB	3	RLK (WAK)	1
6HLH	1	RLK (RLCK)	1
NF-YB	1	CAMK	1

### Protein–protein interaction network

**Figure 4:**
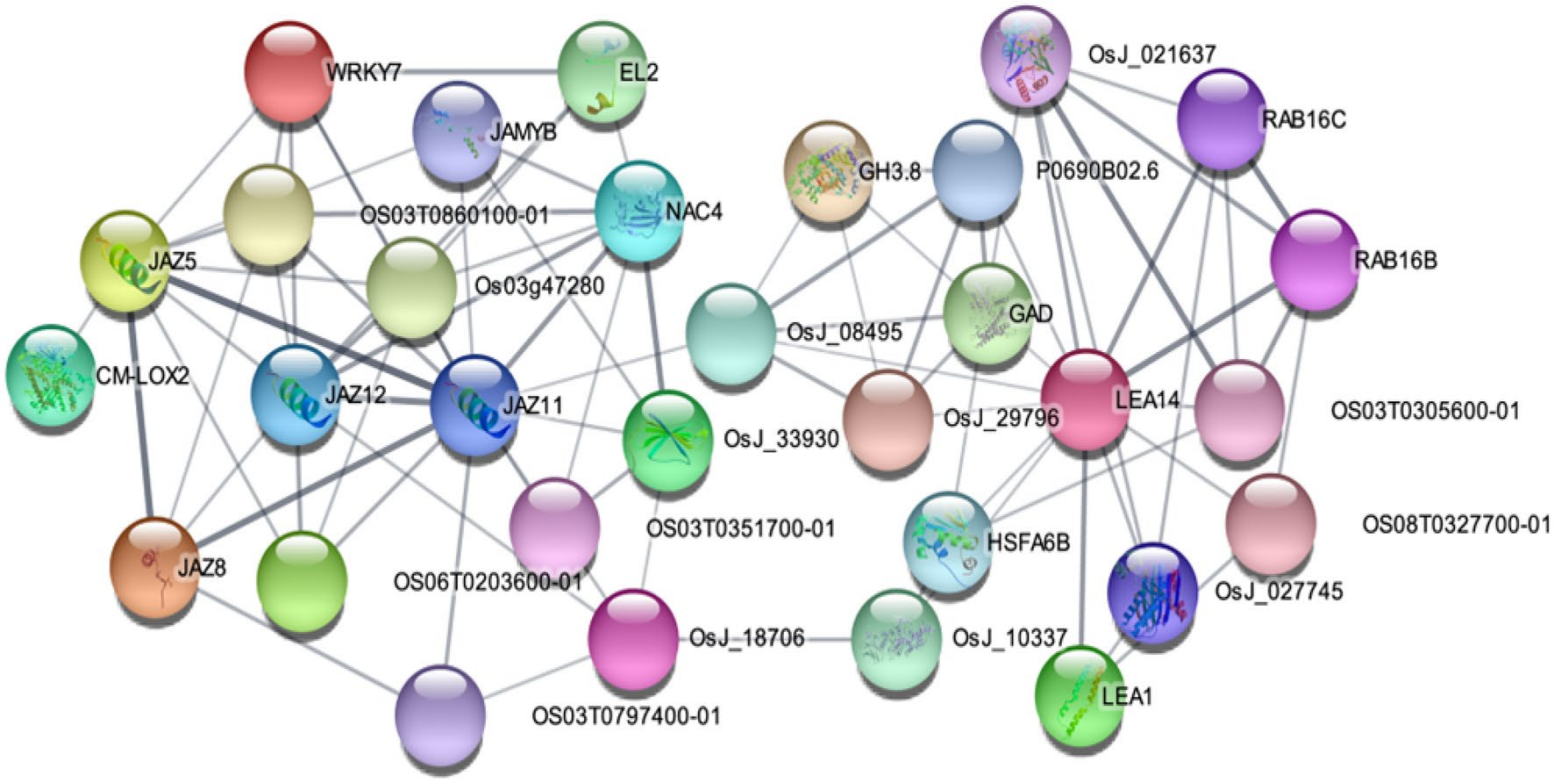
Protein–protein interaction network of hub genes in response to multiple abiotic stresses conducted by Cytoscape 3.8.2 based on MCC method.

The network of hub proteins is shown in Fig. 4. In this network, a total of 31 hub proteins interacted, which the stress response proteins such as JAZ, LEA, NAC, RAB, and WORKY families had the highest interaction in response to abiotic stresses. The top 12 hub proteins are represented in Table S3. The highest interaction scores were related to proteins LEA14, HSFA6B, RAB16B, OsJ_021637, RAB16C, and OS03T0305600-01with scores of 164, 142, 133, 133, 130 and 129, respectively (Table S3). The complete images of the gels are available in the Fig. S5.

### WGCN analysis

**Figure 5:**
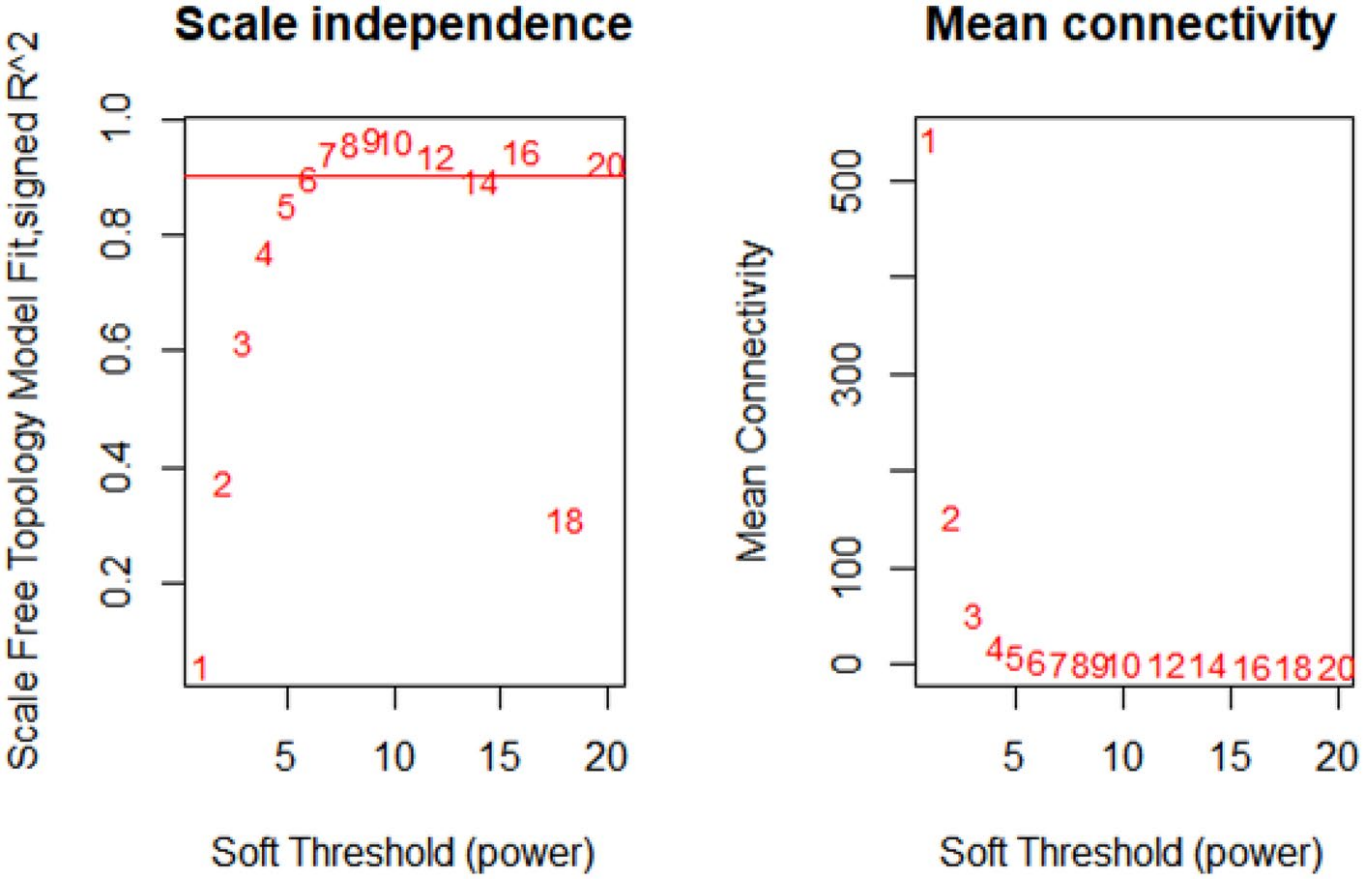
Analysis of network topology for various soft-thresholding powers. The left panel shows the scale-free fit index (y-axis) as a function of the soft-thresholding power (x-axis). The right panel displays the mean connectivity degree (y-axis) as a function of the soft-thresholding power (x-axis).

**Figure 6:**
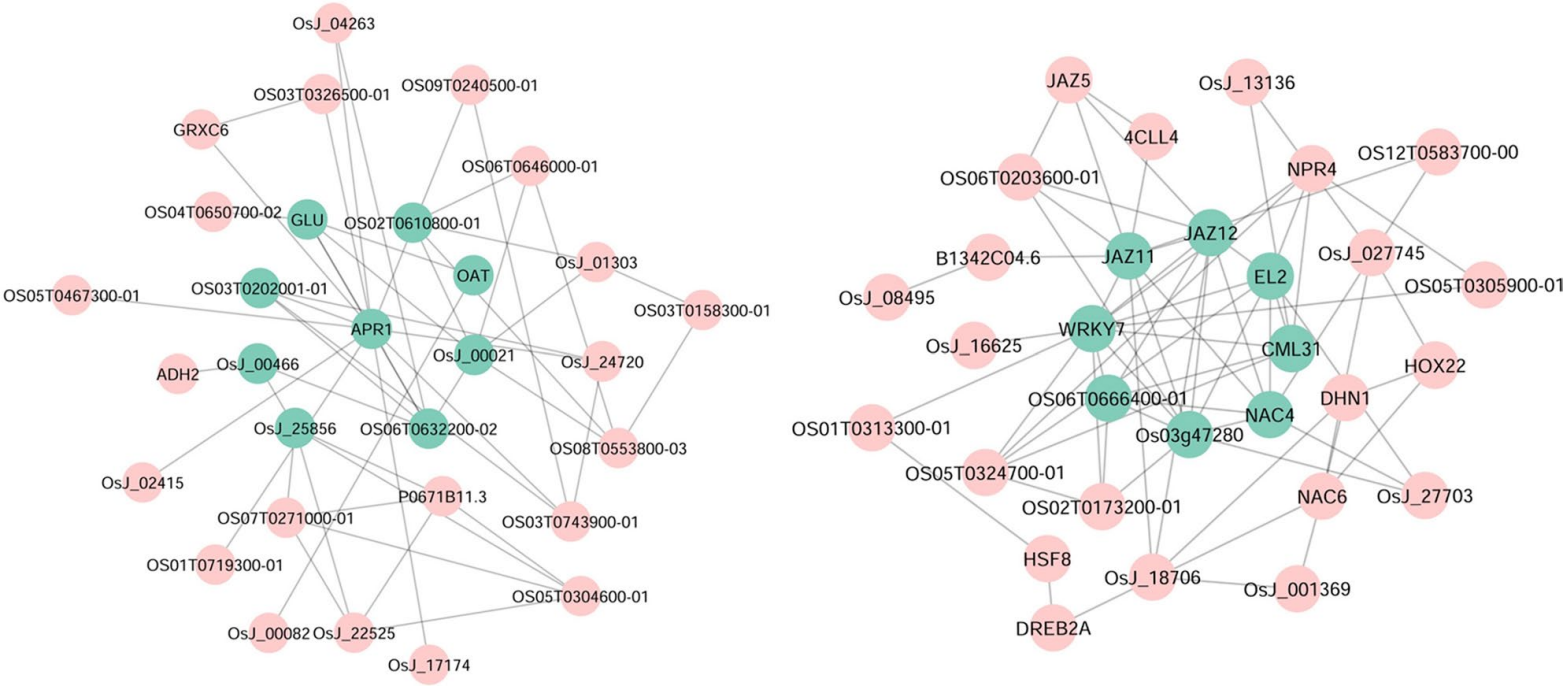
Gene network of the two largest modules conducted by Cytoscape 3.8.2. (**a**) Turquoise module. (**b**) Blue module. Genes with high connectivity are shown in green.

A total of 17 WGCNA modules were identified based on the dynamic tree-cutting algorithm (Fig. S2). The number of genes in each module varied from 57 to 483. The turquoise (483 genes), blue (316 genes), brown (248 genes), and yellow (244 genes) were four major modules. As shown in Fig. 5, when the soft threshold power is defined as 6, the scale-free topological index is 0.9. Therefore, the network is closer to the real biological network state as it adheres to the power-law distribution. Results showed that the six modules (turquoise, blue, yellow, pink, magenta, and tan) were directly involved in abiotic stress processes. Turquoise and blue were the largest modules in the gene networks (Fig. 6). The most important genes in stress response pathways were the JAZ, WPKY, NAC, APR1, and GLU families in the networks.

### Validation of DEGs using qRT-PCR

The specificity of the designed primers (Jasmonate-ZIM domain-containing protein 5 (*JAZ5*) also known as *TIFY9, RAB16B,* actin depolymerizing factor 3 *(ADF3),* Os01g0124650*,* and Os05g0142900*)* was evaluated by agarose gel electrophoresis 1% (Fig. S3), which no non-specific band and primer dimer were observed. To validate the result, the expression of these genes was evaluated by qRT-PCR in all of the studied stresses using *elF1α* as the reference gene to normalize CT values.

The ANOVA analysis of different DEGs in Rice that grew under abiotic stresses indicated that the single effects of stress treatments were highly significant (p < 0.01) in TIFY9, ADF3, Os01g0124650, RAB16B, and Os05g0142900 (Table 5).

**Table 5:** ANOVA Analysis of Some DEGs in rice under multiple abiotic stresses.

Sources of variation	df	TIFY9	ADF3	Os01g0124650	RAB16B	Os05g0142900
Stress	12	6.4**	5.7**	2.5**	7.2**	8.1**
Error	24	0.003	0.002	0.004	0.002	0.001

**Significance at P < 0.01.

The *TYFY9* was significantly upregulated in all stresses except potassium deficiency for 6 h (K-6h), in which a non-significant decrease was shown. The highest expression of *TYFY9* was observed under Iron toxicity, followed by Nitrogen starvation, cold (Exposure of plants to a temperature of 4 °C for 48 h), alkali situation, and potassium deficiency for 5 days (K-5d). The *TYFY9* was highly upregulated in K-5d (log2FC = 5) but downregulated in K-6h (log2FC = − 0.88).

**Figure 7:**
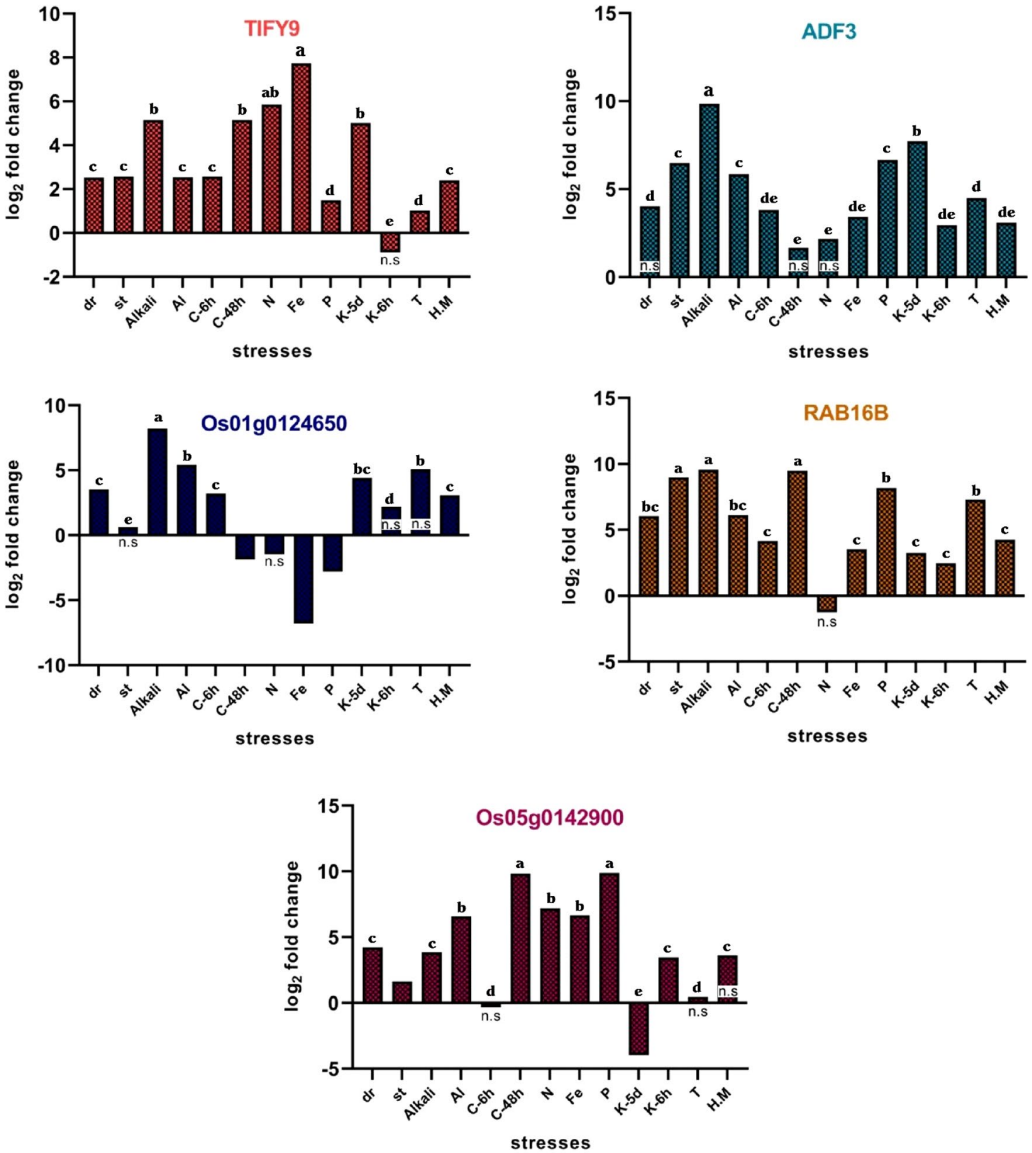
Validation of selected genes using qRT-PCR in different stresses. dr: drought stress/st: salinity stress/Alkali: alkali condition/Al: aluminum toxicity/C-6h: cold exposed for 6 h/C-48h: cold exposed for 48 h/N: nitrogen deficiency/ Fe: Fe toxicity(Iron toxicity)/P: phosphor deficiency/K-5d: Potassium deficiency for 5 days/K-6h: Potassium deficiency for 6 h/T: high temperature/H.M: excess heavy metal (K2Cr2O7). Expressions with not significant changes are represented by n.s, other expressions are significantly up (positive data) or down (negative data) regulated.

The expression of ADF3 increased in all stresses but was not significant under drought, cold-48h, and N starvation conditions. The highest expression of ADF3 was observed under alkali situations (log2FC = 9.8), K-5d (log2FC = 7.72), phosphorus deficiency ((log2FC = 6.65), salinity condition (log2FC = 6.47) and aluminum toxicity (log2FC = 5.84). The *Os01g0124650* was significantly upregulated in six stresses including drought and alkali situation, aluminum and heavy metal toxicity as well as cold conditions for 6 h (C-6h) and potassium deficiency for 5 days (K-5d). Although this gene was upregulated in K-6h, high temperature (T), and salinity conditions, it was not statistically significant. The *RAB16B* was significantly upregulated in all the stresses except for N starvation, in which gene expression was equal to − 1.25 (significant downregulation). The highest expression of *RAB16B* was shown in the C-48h, alkali situation, salinity stress, phosphorus deficiency, and high temperature (T). The *Os05g0142900* had expression levels of 9.87, 9.82, 7.17, 6.66, and 6.58 under phosphorus deficiency, C-48h, nitrogen starvation, Fe, and aluminum toxicity stresses, respectively. Significant downregulation of this gene was observed under K-5d. In general, the qRT-PCR results highly confirmed the outcome of the meta-analysis (Fig. 7) although some treatments had contradictory results.

## Discussion

A meta-analysis was conducted to identify genes responsible for a wide range of abiotic stresses including drought, salinity, cold and high temperatures, alkali conditions, iron, aluminum, heavy metal toxicity, and nitrogen, phosphorus, and potassium deficiency. Plants change their transcriptome profile to endure unfavorable environmental conditions in response to different stresses^42,43^. Despite having a specific response to each stress in plants, this study indicates that there are a vast number of genes that are similarly expressed in response to different abiotic stresses, indicating that there is a core response system to all environmental stresses in rice. Our meta-analysis investigated 11 different abiotic stresses to find the central network of response in rice. Our study identified 561 DEGs, in which 422 genes were upregulated and 139 genes were downregulated (Table S1). Our mata-analysis approach was validated by real-time PCR, and the expression results mainly confirmed it. The TIYF9 protein has previously been identified that regulating defense response and signaling pathway mediated by jasmonic acid in response to wounding in rice^44^. The Os01g0124650 is a family of serine-type endopeptidase inhibitors, which are known as Bowman-Birk inhibitors (BBI). They modulate endogenous proteolytic activities in different developmental stages and prevent exogenous proteases as an element of defense mechanisms in plants^45^. The BBI may also be engaged in multiple abiotic stress responses apart from their important role in defense against pathogenesis^45^. Shan et al. showed that the expression of WRSI5 increased in a salt-tolerant wheat cultivar under salt, drought, or oxidative stresses^46^.

Actin-depolymerizing factor family (ADF3) has been recognized as a stress-responsive protein^47^. It has been reported that overexpression of *OsADF3* in *Arabidopsis* increased the tolerance to drought/osmotic stress by regulating some downstream responsive genes to abiotic stress^48^. A proteomics analysis of rice in 2006 showed a high level of ADFs expression in drought-tolerant cultivars^49^. The Rab proteins belong to the small G protein family, which are involved in different activities including intracellular signaling events, vesicle trafficking, various physiological processes, and stress response^50^. It has been reported that Dehydrin Rab16B is involved in response to abscisic acid, water deprivation, and cold acclimation^44^. Rab16 has been known as an abscisic acid (ABA)-responsive gene, which can sense the ABA and induce downstream stress signaling responses^51^. The Os05g0142900 is an unknown gene, and its expression was determined significantly high under different stresses when it was compared with the control treatment, suggesting a novel gene in response to abiotic stresses. Therefore, a functional analysis is needed to elucidate the potential function of this gene in response to stress.

In our study, the most enriched GO terms in biological processes were "response to stress" and "response to stimulus". Enriched terms in molecular function were "catalytic activity", "oxidoreductase activity", "DNA binding", and "transcription regulator activity". Among the stress-responsive genes, transcription factors are very important because the expression of other stress-responsive genes is regulated by their products by attaching to regulatory elements^52^. Ethylene response factor (ERF) and WRKY family (WRKY23, WRKY30, WRKY50, WRKY56, WRKY70, and WRKY71) had the highest number of genes among the enriched transcription factors in our study. The ERFs are a large subfamily of APETALA2 (Ap2) and are known for their Ap2 domain^53^. Ethylene is essential for many developmental processes and responds to biotic and abiotic stresses^53^. Recent reports have shown that different ERFs attach to dehydration-responsive factors (DREs) and act as key regulatory factors in plant responses to abiotic stresses^54^. It has been reported that the expression of ERF increased under drought, salinity, light, cold, and high-temperature stresses^53^. Jasmonic acid and abscisic acid are also involved in regulating the expression of ERFs under abiotic stresses^55^. The ethylene signaling pathway is also interconnected with other phytohormone pathways, which are regulated by salicylic acids, gibberellins, and brassinosteroids when plants are adapted to abiotic stresses^55^. Previously it was reported that the application of exogenous phytohormones also increased the expression of ERF genes^55^.

The WRKY proteins have an important function in cellular metabolism including the biosynthesis of phytohormones, phytoalexins, and other chemicals engaged in cellular defense^56^. The WRKY transcription factors regulate plant growth^57^ and play an important role in plants' responses to biotic and abiotic stresses^58–60^. The WRKY may correlate biotic stress-responsive proteins and abiotic stress-responsive proteins^58^. In our study, vesicle groups and cytoplasmic and vesicular membranes had the highest number of DEGs among cellular components. In plants, endomembrane trafficking is an essential mechanism that responds to environmental stresses^61,62^. Plants as immobile organisms continuously monitor environmental changes to be capable of altering their metabolism and gene expression profile in response to shifted conditions^63^. It has been widely indicated that plants have an effective response system to deal with stresses including the primary and secondary perception of stress and signal transmission in cells^64^. Early perception of stress occurs with changes in membrane leucine-rich receptors^65^. In addition, secretory pathways regulated by vesicular walls act in the stress tolerance mechanism. The first stage of the secretory pathway is regulated by coat *protein* II from the vesicular and induces the transfer of cargo, which usually are stress-damaged proteins, from the endoplasmic reticulum to the Golgi apparatus^66^.

The KEGG result showed that DEGs were mostly involved in "metabolic pathways", "biosynthesis of secondary metabolites", "transport of plant hormones", "biosynthesis of phenylpropanoids, nucleotide, and amino acids glucose metabolites" and "MAPK signaling pathway." It has been previously reported that plant metabolites protect plants against high salinity and drought stresses^67^. Metabolites such as glutathione, ascorbic acid, anthocyanins, tocopherols, and carotenoids protect plants from oxidative damage associated with different stresses by inhibiting reactive oxygen species (ROS) production during oxidative stress. Jasmonic acid, methyl jasmonate, salicylic acid, methyl salicylate, and other small molecules produced during stress conditions can also be activated as signaling molecules to induce defense responses and lead to a systemic adaptation^67^. Mitogen-Activated Protein Kinase (MAPK) are highly conserved signal transmission modules and are involved in in many signal transmission processes through the MAPK cascade^68^. The messages were transmitted from the extracellular into the cell by activating downstream kinases, enzymes, and transcription factors^69^. Recently it was reported that elevated expression of genes associated with the MAPK pathway increased the resistance to stress in crops^70^.

Our findings have identified candidate genes associated with various abiotic stresses, which can be further investigated to understand the core mechanisms underlying the response of rice to multiple stress conditions. Many individual studies have been done to investigate the response of rice to stresses but our meta-analysis covers a wide range of abiotic stresses. The meta-analysis approach can be used to study different plants' mechanisms in different situations, particularly for plants with less information. The current study includes abiotic stresses, suggesting that further research should concentrate on environmental and biological stresses for a better understanding of the relationship between abiotic and biotic response systems in plants.

### Supplementary Information

Supplementary Figures.

Supplementary Tables.

## Data Availability

The datasets analyzed during the current study are available in the [NCBI] repository, [GEO Datasets] with accession numbers of GSE14275, GSE93917, GSE3053, GSE107531, GSE45724, GSE37940, GSE109649, GSE37161, GSE60823, GSE131287, and GSE25206.
