# Supplementary material for: Identification of responsive genes to multiple abiotic stresses in rice (Oryza sativa): a meta-analysis of transcriptomics data
Source: Sci Rep. 2024 Apr 1;14:5463. doi: 10.1038/s41598-024-54623-7 (PMC10985071; doi:10.1038/s41598-024-54623-7)
Supplement: Supplementary file 1 — Supplementary Figures. [file 41598_2024_54623_MOESM1_ESM.docx]

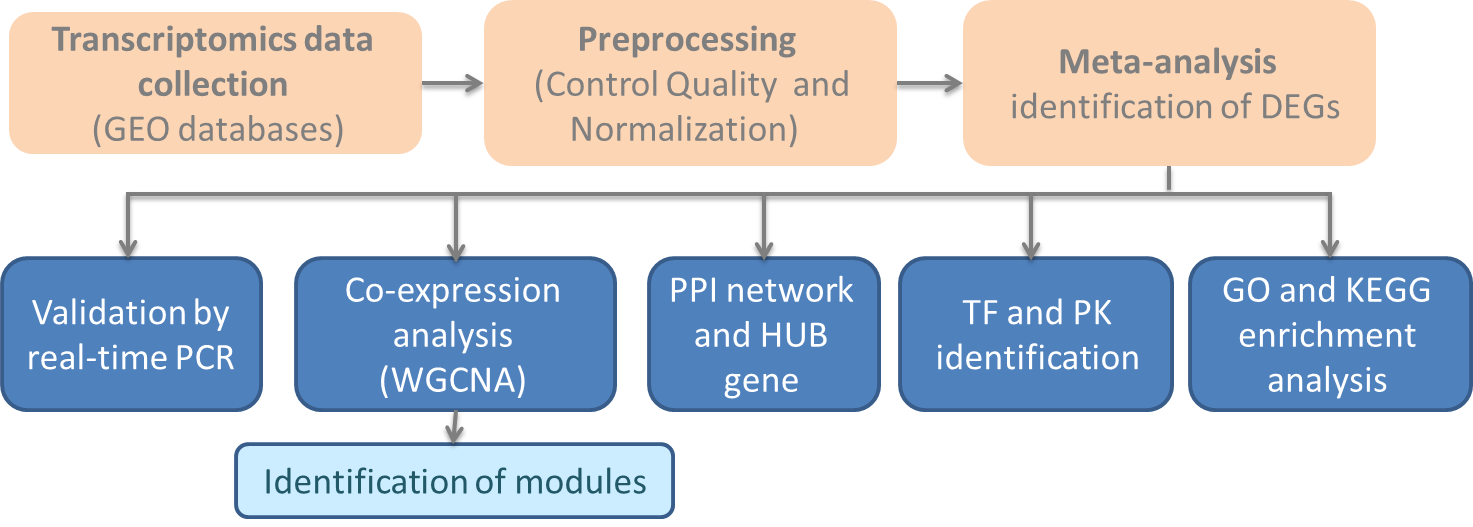
 Figure S1. Schematic overview of the analysis process to investigate the response of rice (*Oryza sativa*) to multiple abiotic stress.


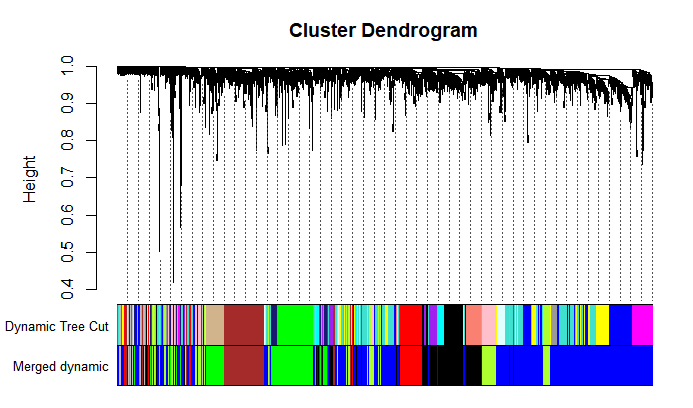
 Figure S2. WGCNA module identification and correlation analysis. Gene dendrogram obtained by clustering the dissimilarity based on consensus Topological Overlap with the corresponding module colors indicated by the color row. Each colored row represents a color-coded module that contains a group of highly connected genes. A total of 17 modules were identified.


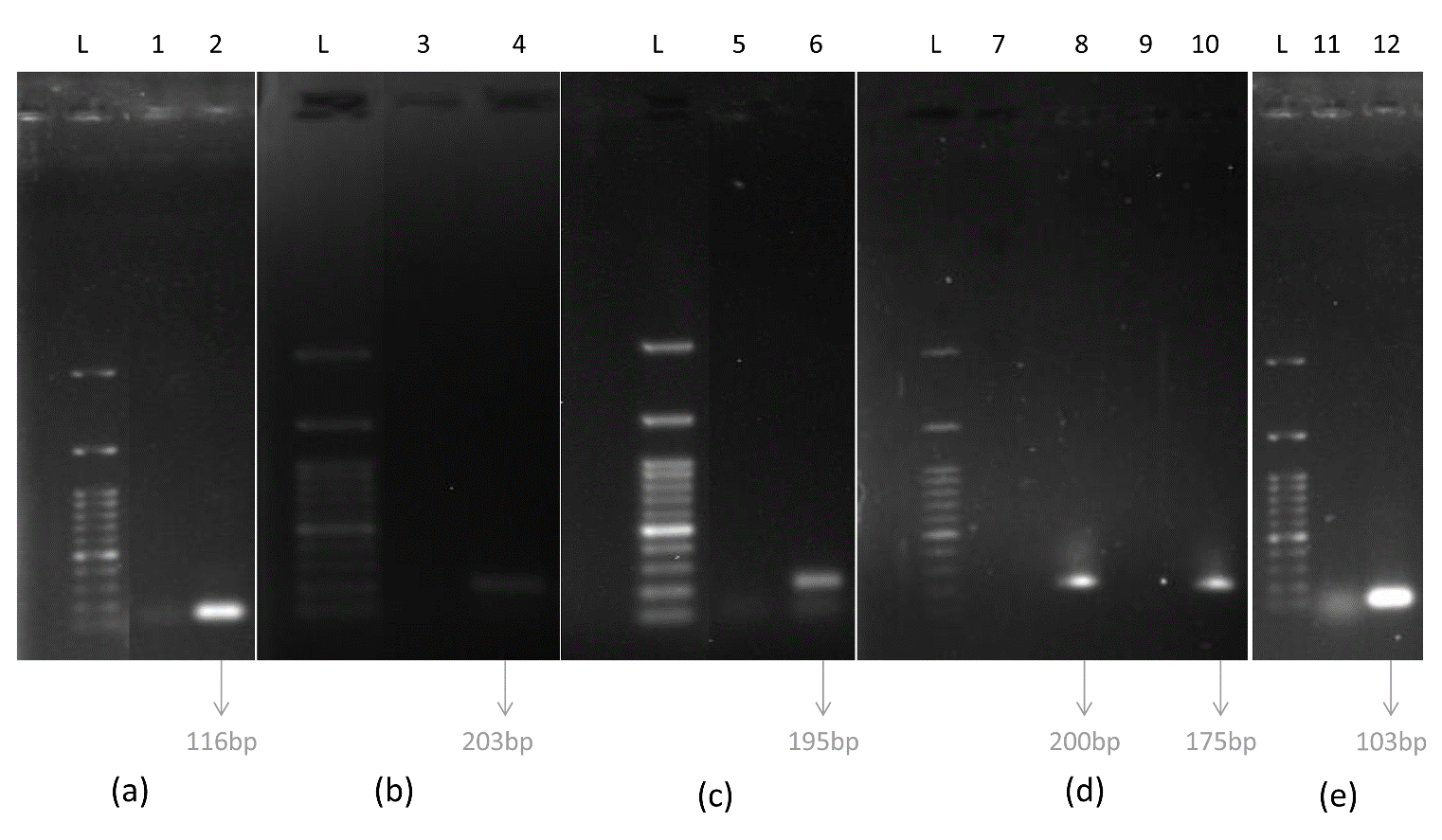
 Figure S3. Agarose gel electrophoresis (1%) for PCR products of different genes. L: DNA ladder. (a). Lane 1: negative control of *Os01g0124650* gene, Lane2: *Os01g0124650* gene; (b). Lane 3: negative control of *Os05g0142900* gene, Lane4: *Os05g0142900* gene; (c). Lane 5: negative control of *TIFY9* gene, Lane6: *TIFY9* gene; (d). Lane 7: negative control of *ADF3* gene, Lane8: *ADF3* gene, Lane 9: negative control of *RAB16B* gene, Lane10: *RAB16B* gene; (e). Lane 11: negative control of *elF1α* gene, Lane12: *elF1α* gene. Size of the bands is indicated in the picture. Band sizes of ladder from top to bottom are 3000, 1500, 1000, 900, 800, 700, 600, 500, 400, 300, 200 and 100bp respectively. The ladder map is available in Fig S4. The gel images are cropped for ease of display, and the full images of the gels are provided in Fig S5.


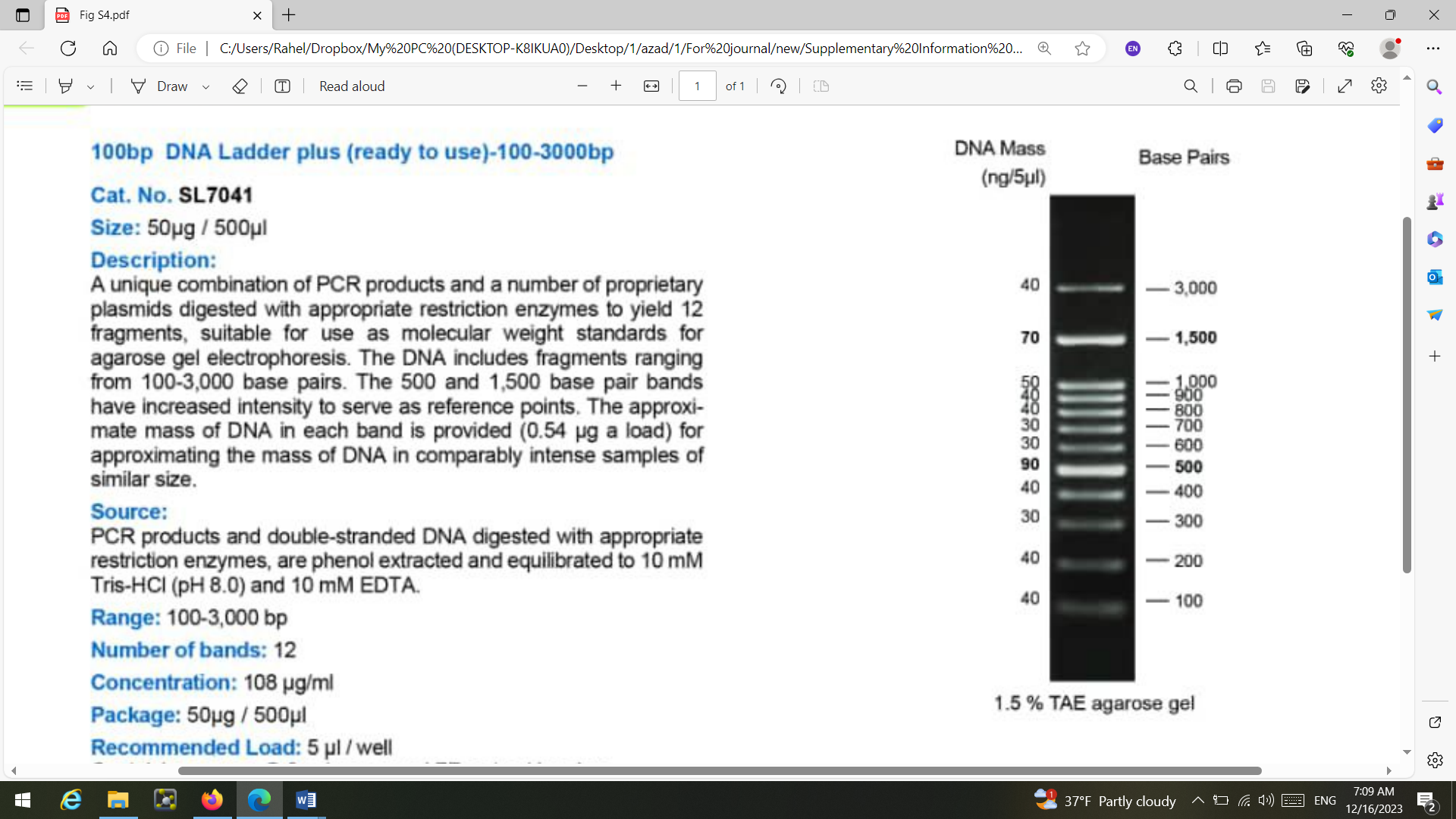


Fig S4. DNA Ladder map used in this experiment


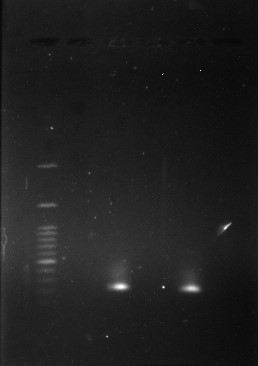


ADF3,RAB16B-lane7-10


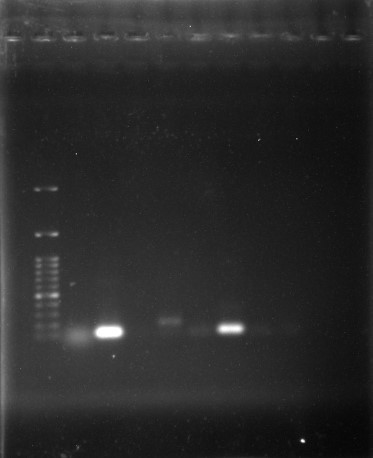


ELF-lane 1&2


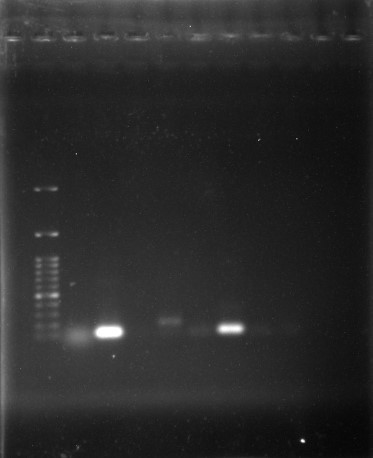


Os01g0124650-lane 5&6 after ladder


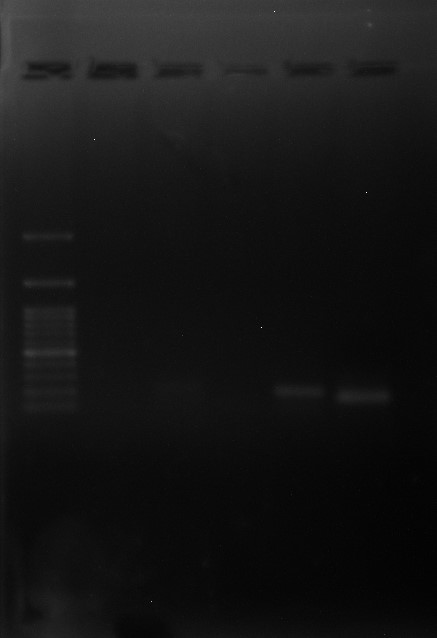


Os05g0142900-4&5 lane


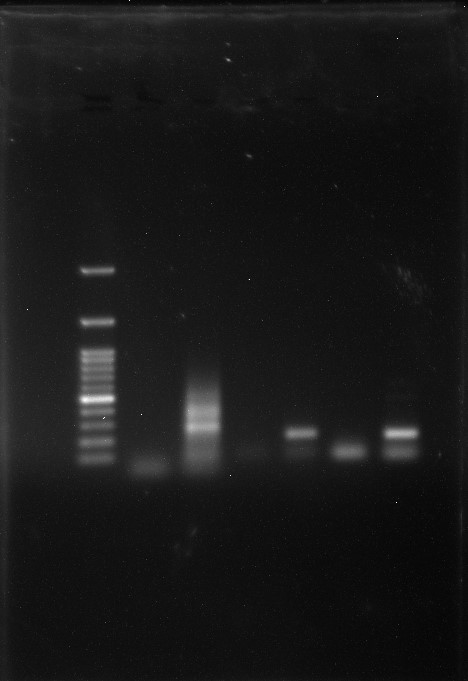


TIFY9-3&4 after ladder

Fig S5. Full image of the gels presented inside the article
